# Brain Abscess Caused by Listeria monocytogenes: A Rare Case of Supratentorial Neurolisteriosis

**DOI:** 10.7759/cureus.54521

**Published:** 2024-02-20

**Authors:** Mafalda Vasconcelos, Ana Patrícia Moreira, Carla Sofia Pereira, Rui Duarte Armindo, Carla Noronha

**Affiliations:** 1 Internal Medicine, Hospital Beatriz Ângelo, Lisbon, PRT; 2 Neuroradiology, Hospital Beatriz Ângelo, Lisbon, PRT

**Keywords:** foodborne pathogens, immunocompromised individuals, neurolisteriosis, brain abscess, listeria monocytogenes

## Abstract

*Listeria monocytogenes* is a Gram-positive bacillus that presents a tropism for the central nervous system (CNS). In fact, CNS involvement occurs in over two-thirds of infections caused by this agent. Meningitis is the most common manifestation, while brain abscess is rare.

We present the case of a 77-year-old male patient on corticosteroid treatment for bronchiolitis obliterans organizing pneumonia with a history of unpasteurized cheese consumption, who presented with fever and altered mental status. Brain computerized tomography scan revealed left frontal cortico-subcortical hypodensity mimicking an ischemic stroke. Subsequent magnetic resonance imaging revealed a brain abscess, and blood cultures yielded *Listeria monocytogenes*. A good clinical outcome was achieved after appropriate antimicrobial therapy and abscess drainage.

This case underscores the importance of considering *Listeria monocytogenes* in CNS infections, especially in immunocompromised individuals over 65 years of age. The atypical supratentorial involvement challenges the more common rhombencephalitis presentation.

Maintaining a high level of suspicion in relevant populations is crucial for timely diagnosis and intervention, especially in patients with comorbidities, who present particularly high mortality rates.

## Introduction

*Listeria monocytogenes*, a Gram-positive rod, is primarily transmitted through the consumption of contaminated food. The estimated incidence of listeriosis ranges from 3 to 6 cases per million inhabitants annually in industrialized countries [1]. In 2013, it was the leading cause of hospitalization and death attributed to contaminated food consumption in Europe, with a case fatality rate of 15.6% (among the 1,228 cases with known outcomes out of a total of 1,763 confirmed cases) [2].

Unlike many other foodborne pathogens, *Listeria monocytogenes* can thrive in food with low moisture content, high salt concentration, and refrigeration temperatures, posing a considerable challenge for control measures. Transmission occurs mainly through the ingestion of unpasteurized dairy products or contaminated meat and seafood [3].

Clinical manifestations of listeriosis predominantly affect at-risk groups, including pregnant women, elderly individuals, immunocompromised individuals, fetuses, and neonates [4]. In healthy individuals, the infection typically results in self-limiting febrile gastroenteritis. However, in immunocompromised individuals, listeriosis can progress to severe illnesses [5].

In at-risk patients, listeriosis often presents invasively with three major clinical forms, namely, bacteremia, central nervous system (CNS) infection, and maternal-fetal listeriosis. Among non-pregnant adults, CNS involvement constitutes the most prevalent clinical form, occurring in 55% to 70% of cases, with known predisposing factors being age over 50 years and conditions such as malignancy and diabetes [5,6].

## Case presentation

We describe the case of a Portuguese caucasian 77-year-old man who presented to our hospital’s emergency department with a two-month history of altered mental status, aggravated in the previous week.

He had a known history of arterial hypertension, dyslipidemia, diabetes mellitus, and atrial fibrillation on oral anticoagulants. Three months earlier, he had been admitted to a different hospital with shortness of breath. After investigation, a diagnosis of bronchiolitis obliterans organizing pneumonia was established and confirmed on pulmonary biopsy. Since then, the patient had been on corticosteroids (initially prednisolone 1 mg/kg/day with subsequent tapering), with satisfactory resolution of respiratory symptoms. One month earlier, after hospital discharge, he presented with insomnia, agitation, and hetero-aggressive behavior, symptoms that were attributed to corticosteroid-induced psychosis and led to a more rapid tapering of prednisolone down to 2.5 mg/day.

On clinical examination, the patient was febrile and hypotensive, with a productive cough and inspiratory crackles on the left pulmonary basis. On neurological examination, he presented with an altered mental status with drowsiness, disorientation, mild inattention, difficulty following complex commands, and anomic pauses. There were no meningeal signs or other neurologic deficits.

**Figure 1:**
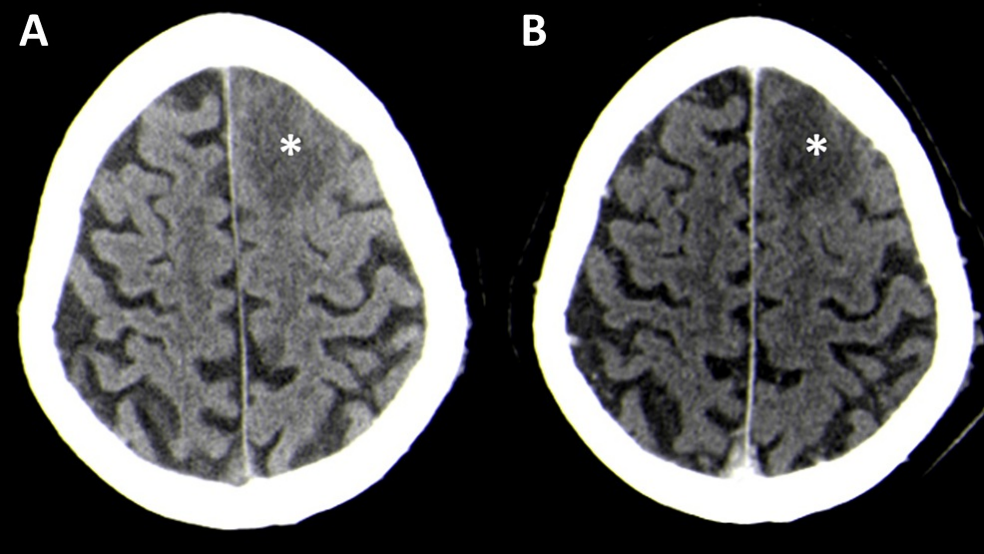
Head computerized tomography pre-contrast (A) and post-contrast (B) axial images at hospital admission showing a left frontal cortico-subcortical hypodensity (asterisks) without significant enhancement after contrast.

Initial workup revealed normocytic normochromic anemia (hemoglobin 10.7 g/dL) and mild C-reactive protein elevation (8 mg/dL) with normal leukocyte count. Arterial blood gas showed no respiratory insufficiency. Chest computerized tomography (CT) scan showed bilateral ground-glass opacities, without other significant findings. Severe acute respiratory syndrome coronavirus 2, influenza A and B, and urinary antigen testing for *Streptococcus pneumoniae* and *Legionella pneumophila* were negative. A head CT scan showed a left frontal cortico-subcortical hypodensity of suspected ischemic etiology (Figure 1).

Lumbar puncture revealed mild pleocytosis (18 cells) with a predominance of mononuclear cells, a slight increase in protein concentration (55 mg/dL), and a normal glucose level. Cerebrospinal fluid (CSF) cultures were negative. Blood cultures were positive for *Listeria monocytogenes* (antibiotic susceptibility testing showing susceptibility to all the antibiotics tested, namely, penicillin, amoxicillin, trimethoprim-sulfamethoxazole, erythromycin, and meropenem). The patient was started on ampicillin and gentamicin. Venereal Disease Research Laboratory test and human immunodeficiency virus, hepatitis B, and hepatitis C serologies were negative. On further questioning, he admitted to the recent consumption of significant amounts of cheese confectioned with unpasteurized milk.

**Figure 2:**
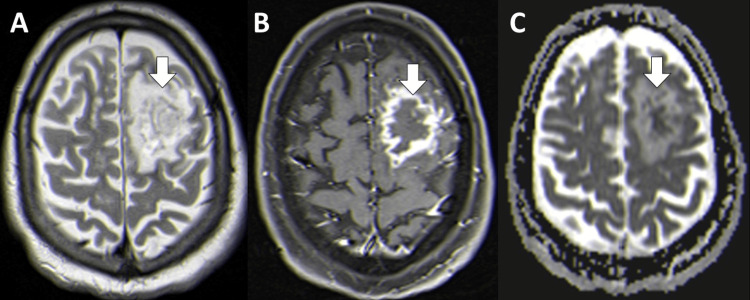
Brain magnetic resonance axial images evidencing a left frontal subcortical mass lesion (arrows) with heterogeneous high signal on T2-weighted imaging (A), a thin enhancing rim after contrast (B), and central restricted diffusion evident on the apparent diffusion coefficient map (C).

To better characterize the lesion observed on the CT scan, brain magnetic resonance imaging (MRI) was performed, showing a large left frontal subcortical space-occupying lesion with a thin enhancing rim after contrast and central restricted diffusion on diffusion-weighted imaging/apparent diffusion coefficient, compatible with a brain abscess (Figure 2). The abscess was surgically drained and *Listeria monocytogenes* was isolated from the cultures of the purulent material.

**Figure 3:**
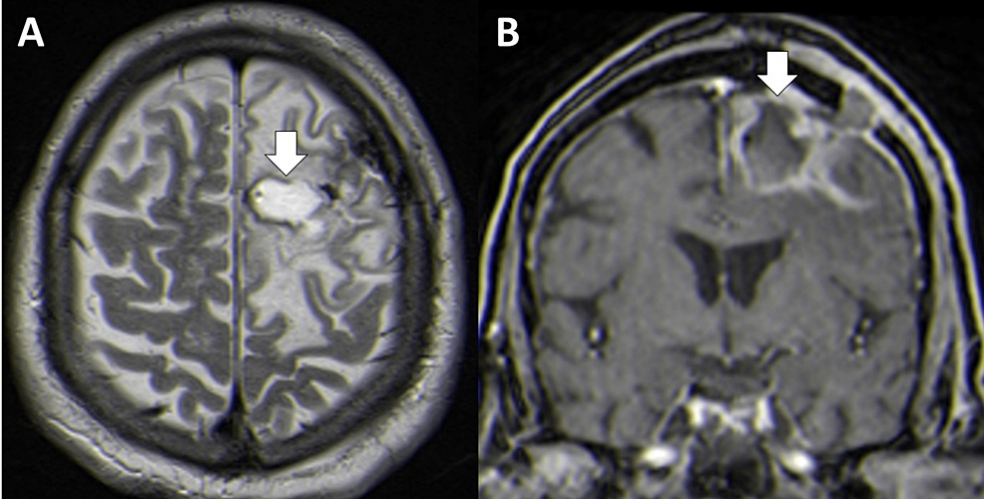
Follow-up brain magnetic resonance imaging: axial T2 (A) and coronal post-contrast T1 (B) images, three weeks after surgery, showing a residual encapsulated abscess (arrows) with perilesional vasogenic edema.

The patient evolved favorably, with sustained apyrexia and recovered mental status, maintaining solely rare anomic pauses and phonological paraphasias, for which he was referred to a speech therapy program. Control brain MRI, three weeks after surgery, showed a residual encapsulated abscess with perilesional vasogenic edema (Figure 3), control blood cultures were negative, and a second lumbar puncture revealed resolution of the previously described CSF changes. He was discharged after completing 60 days of ampicillin and 30 days of gentamicin.

## Discussion

*Listeria monocytogenes* exhibits a tropism for both the brain parenchyma and the meninges. Meningitis is the most prevalent CNS infection caused by this pathogen [7]. This was evident in the Multicentric Observational NAtional Study on LISteriosis and ListeriA (MONALISA), a nationwide prospective study in France involving 252 patients with CNS listeriosis [8], where 84% presented with meningoencephalitis. *Listeria monocytogenes* is the primary contributor to bacterial meningitis in specific groups of immunosuppressed individuals, such as patients undergoing corticosteroid therapy and organ transplant recipients [7].

A distinctive manifestation of *Listeria monocytogenes* CNS infection is rhombencephalitis, an acute brainstem and cerebellum infection that can lead to asymmetric cranial nerve palsies and other focal neurologic deficits, cerebellar dysfunction, altered consciousness, and has the potential to evolve to respiratory failure [9].

The occurrence of macroscopic brain abscesses is relatively uncommon, being present in around 10% of listerial CNS infections [10]. The involvement of the subcortical gray matter, in locations such as the thalamus and basal ganglia, is more prevalent in listerial brain abscesses than abscesses arising from other causes [7]. Around a quarter of listerial brain abscess cases exhibit concomitant meningitis caused by the same agent (isolated in CSF cultures) [11]. The presence of bacteremia is nearly universal, supporting the hypothesis that the development of listerial brain abscesses is a consequence of hematogenous spread rather than spread from adjacent tissues [7].

When there is suspicion of CNS listeriosis, CSF analysis, culture, and polymerase chain reaction (PCR) should be performed, along with blood cultures. CSF may only reveal mild abnormalities or pleocytosis, with the predominance of lymphocytes. Gram stain tends to be negative but the cultures are usually positive for *Listeria monocytogenes*, even when there are only mild alterations in the CSF. When CSF examination is not conclusive, it may be associated with the formation of an abscess capsule [12]. The diagnosis can be obtained directly when CSF cultures or PCR are positive, or when blood cultures are positive and neurological clinical findings point to the disease. In this case, imaging is essential, with MRI with contrast being the gold standard [9].

The commonly prescribed antimicrobial regimen involves the combination of ampicillin and an aminoglycoside. Ampicillin administration should extend for a minimum of four weeks, while gentamicin is typically given for two to four weeks [7]. For individuals allergic to penicillin, trimethoprim-sulfamethoxazole may serve as a reasonable alternative antibiotic. Cephalosporins are generally not recommended, as *Listeria monocytogenes* often exhibits partial or complete resistance to them. In cases with localized CNS involvement, prolonged antibiotic therapy is necessary, lasting at least five to six weeks. Patients should undergo serial neurological imaging, with MRI being the preferred modality [11].

Recommendations for surgical aspiration include abscesses exceeding 2.5 cm in size, those situated in deep brain regions (such as the cerebellum or diencephalon), and cases where identification of the infective agent is necessary to guide appropriate therapeutic interventions [13].

Mortality associated with *Listeria monocytogenes* infection is influenced by the severity of the infection, presence of immunodepression, timely diagnosis, and early initiation of treatment. Risk factors for mortality due to listeriosis are age over 60 years, primary bacteremia, and CNS involvement [14]. In the MONALISA study, overall three-month mortality was 46% for patients presenting with bacteremia and 30% for neurolisteriosis [8].

The differential diagnosis for* Listeria* CNS involvement includes infections caused by other bacterial agents (such as *Streptococcus pneumoniae*, *Neisseria meningitidis*, or *Mycobacterium tuberculosis*), viruses (including herpes simplex, varicella-zoster, and cytomegalovirus), fungi (such as *Cryptococcus neoformans* or *Aspergillus* spp.), or parasites (such as *Toxoplasma gondii* or *Taenia solium*). Apart from stroke, other non-infectious conditions should be considered, such as malignancies (metastatic brain tumors or primary CNS tumors, including CNS lymphoma), and inflammatory or autoimmune conditions (such as multiple sclerosis or vasculitis).

## Conclusions

This case highlights the importance of considering *Listeria monocytogenes* as a potential causative agent in patients with CNS involvement, particularly in immunocompromised individuals and those over 60 years of age. While infections caused by this agent are relatively uncommon, they can lead to severe and particularly rare CNS manifestations, such as brain abscesses, which may initially mimic other neurological conditions, such as ischemic strokes.

The effective treatment of neurolisteriosis can be challenging, often necessitating prolonged antimicrobial therapy and surgical intervention for larger or deep-seated abscesses. Early diagnosis and treatment initiation are vital to achieving a favorable outcome, given the high mortality rate associated with CNS involvement, especially in patients with comorbidities. For this reason, a high index of suspicion for this agent should be maintained.
